# Recent advances on the molecular mechanisms of exercise-induced improvements of cognitive dysfunction

**DOI:** 10.1186/s40035-023-00341-5

**Published:** 2023-02-27

**Authors:** Yi Lu, Fa-Qian Bu, Fang Wang, Li Liu, Shuai Zhang, Guan Wang, Xiu-Ying Hu

**Affiliations:** West China School of Nursing, Sichuan University/Innovation Center of Nursing Research, Nursing Key Laboratory of Sichuan Province, State Key Laboratory of Biotherapy and Cancer Center, National Clinical Research Center for Geriatrics, West China Hospital, Sichuan University, Chengdu, 610041 China

**Keywords:** Exercise, Cognitive function, Molecular mechanism, Neurodegenerative disease, Nonpharmacological therapy

## Abstract

Physical exercise is of great significance for maintaining human health. Exercise can provide varying degrees of benefits to cognitive function at all stages of life cycle. Currently, with the aging of the world’s population and increase of life expectancy, cognitive dysfunction has gradually become a disease of high incidence, which is accompanied by neurodegenerative diseases in elderly individuals. Patients often exhibit memory loss, aphasia and weakening of orientation once diagnosed, and are unable to have a normal life. Cognitive dysfunction largely affects the physical and mental health, reduces the quality of life, and causes a great economic burden to the society. At present, most of the interventions are aimed to maintain the current cognitive level and delay deterioration of cognition. In contrast, exercise as a nonpharmacological therapy has great advantages in its nontoxicity, low cost and universal application. The molecular mechanisms underlying the effect of exercise on cognition are complex, and studies have been extensively centered on neural plasticity, the direct target of exercise in the brain. In addition, mitochondrial stability and energy metabolism are essential for brain status. Meanwhile, the organ-brain axis responds to exercise and induces release of cytokines related to cognition. In this review, we summarize the latest evidence on the molecular mechanisms underlying the effects of exercise on cognition, and point out directions for future research.

## Background

Cognitive decline occurs during the aging process. It is often observed in two populations, the elderly people with mild cognitive impairment (MCI) and cognitive dysfunction, and patients with neurodegenerative diseases [1, 2]. The prevalence of MCI increases with age (6.7% for ages 60–64, 8.4% for 65–69, 10.1% for 70–74, 14.8%  for 75–79, and 25.2% for 80–84) [1]. Cognitive dysfunction is frequently seen in neurodegenerative diseases, and the high incidence of neurodegenerative diseases increases the prevalence of cognitive dysfunction. There were 35.6 million people diagnosed with dementia in 2010 worldwide [3], and this figure is expected to double every 20 years [4], which will reach 65.7 million in 2030 and 150 million in 2050 [5]. At present, the cost of care for current 35 million patients with dementia is over $600 billion per year, accounting for approximately 1% of the global gross domestic product [6]. Apart from dementia, cognitive impairment is also identified in 34%–65% of people with multiple sclerosis (MS), and 40% with Parkinson’s disease (PD) [7, 8].

Neurobiological features of cognitive impairment are specific among different diseases. Brain plasticity deficits are often observed in age-related cognitive impairment. The hippocampus plays a key role in brain plasticity and has a classical trisynaptic circuit. The entorhinal cortex provides the major cortical input to the hippocampus, and the cross-projections between dentate gyrus (DG), cornu ammonis 1 (CA1) and cornu ammonis 3 (CA3) regions are critical for supporting a distributed memory [9]. Therefore, decreases of dendritic branching and synapses in the entorhinal cortex, DG and CA1-CA3 are characteristics of cognitive decline in aged population [10]. It is believed that extracellular aggregation of amyloid beta (Aβ) and intracellular aggregation of tau or formation of neurofibrillary tangles (NFTs) play a critical role in Alzheimer’s type cognitive impairment [11]. Patients with Parkinson’s type cognitive impairment show neuropathology of PD with early loss of dopaminergic neurons in the substantia nigra, and abnormal deposition of α­-synuclein in Lewy bodies initially in cholinergic and monoaminergic neurons in the brainstem and the olfactory system, causing significant synaptic pathology [12]. There is similar neurobiology in other α-synucleinopathies, such as dementia with Lewy bodies (DLB) and multiple system atrophy [13]. For MCI, studies have shown hypoperfusion and hypometabolism in temporoparietal cortices, atrophy of the medial temporal lobe, elevation or phosphorylation of tau, decrease of Aβ42 in the cerebrospinal fluid, and deposition of brain Aβ42 [14]. Despite the differences in neurobiology, the age-related and disease-related cognitive dysfunction are consistent in pathophysiological features, including neuroinflammation, oxidative stress, mitochondrial dysfunction and metabolic alterations [15, 16].

Exercise therapy has been proven effective in promoting cognitive function [17], which can improve mental state such as depression and insomnia stress [18] and delay the progression of neurodegenerative diseases [19]. Different doses and types of exercise may have variable effects. Studies have reported the optimal dose and type of exercise to improve cognition in older adults and provided information that can be directly used to recommend the dose and type of exercise for optimal cognitive health among older adults [20]. A recent systematic review showed that there is no minimal threshold for beneficial effects of exercise on cognition, suggesting that any exercise dose is better than none. Notably, recent evidence also highlights superior effects of resistance exercises over other modalities [20]. Although most research was focused exclusively on aerobic exercise, resistance exercise may also enhance cognition and brain outcomes in older adults [21]. Furthermore, a recent meta-analysis revealed that aerobic exercise combined with resistance training is the most effective to improve cognition among patients with MCI, whereas resistance exercise is the most effective for those with dementia [22]. The mechanisms of the effects of aerobic and resistance training on cognition may be different. For example, Vilela et al. observed increases of BDNF (brain-derived neurotrophic factor), cAMP-response element binding protein (CREB) and p75 neurotrophin receptor after training for both aerobic and resistance exercise in aging Wistar mice. In addition, glutamatergic proteins such as postsynaptic dense substance 95 (PSD-95) and NMDA receptor are increased only in aerobic rats, while resistance training increases levels of protein kinase C (PKC) as well as proinflammatory factors tumor necrosis factor-α (TNF-α) and interleukin-1β (IL-1β) in the hippocampus [23]. Meanwhile, exercise improves cognitive function in older adults in an intensity-dependent manner. Moderate-intensity exercise has been suggested as an optimal intensity for promotion of mental health by decreasing the level of TNF-α [24]. In contrast, strenuous exercise has been shown to enhance inflammation and trigger free radical-mediated damage [25]. Sanguesa et al. observed downregulation of oxidative phosphorylation of complex IV and AMP kinase (AMPK) activation in the cortex of rats after 16 weeks of intense exercise, denoting mitochondrial dysfunction [26].

The molecular mechanisms underlying the effects of exercise on cognitive function have been increasingly investigated. BDNF plays an important role in maintaining cognitive function and protecting nerve plasticity, and it is also an important molecular mediator of the neuroprotective effects of physical exercise [27]. In the pathological process of cognitive disorders, neuronal mitochondrial dysfunction often occurs [28], leading to excessive oxidative stress and energy deficiency in brain tissue. Exercise has been demonstrated to play an important role in improving the stability of neuronal mitochondria [29, 30]. Moreover, exercise can induce skeletal muscle production of myokines into the blood [31], some of which promote expression of BDNF in hippocampus and inhibit production of inflammatory factors [32]. In addition to the direct effects in the brain, such as neuroinflammation and oxidative stress, exercise can also stimulate energy metabolism in the brain [33] and the intestinal-brain axis [34]. In the following, we will summarize the significant and latest molecular mechanisms of the positive effect of exercise on cognitive function from the above-mentioned aspects.

## Exercise enhances brain plasticity

Brain plasticity is the ability of the brain to reorganize its structure and function in response to learning, training and environment stimuli [35]. Brain plasticity includes changes in cortical thickness, gray matter volume, and white matter fiber connectivity strength and orientation at the macroscopic level; neuronal and synaptic morphological changes at the cellular level; changes in intracellular signals and gene transcription within the nucleus after stimulation at the molecular level. The mechanisms involved in plasticity in the nervous system are thought to support cognition [10]. The decline of brain structure and function in the elderly is the main cause of the decline of their cognitive function [36].

Neurotrophic factors and synaptic plasticity are critical mechanisms of brain plasticity. Neurotrophic factors are growth factors that can nourish neurons and promote neuron survival and regeneration [37]. Recent meta-analysis showed that blood levels of BDNF are decreased in PD and MS patients [38, 39]. Another primary functional alteration that could directly affect plasticity is reduced synaptic number, which could make it more difficult to attain sufficient number of cooperatively active synapses, which is necessary for network modification. It is appreciated that MCI in early Alzheimer’s disease (AD) may be due to the synaptic dysfunction caused by accumulation of non-fibrillary, oligomeric Aβ, occurring prior to the evident widespread synaptic loss and neurodegeneration [40].

The maintenance of brain plasticity also depends on the nerve tissue. With progress of age-related neurodegenerative diseases, the number and function of brain nerve tissue begin to decrease, such as reduced phagocytic capacity of microglia, hyperplasia of astrocytes and microvascular changes in the DG of the hippocampus [41]. Recent evidence highlights that neurogenesis in the adult hippocampal body lasts until the tenth decade of human life [42]. Boldrini assessed the anterior, mid, and posterior parts of autopsy hippocampi from 28 healthy individuals (14 to 79 years of age), and characterized and quantified angiogenesis, volume, and cells in the DG neurogenic niche across ages, using unbiased stereological methods. Although the neuroplasticity declines in older individuals, they found that quiescent radial-glia-like type I neural progenitor cells (QNPs) decline, but the number of intermediate neural progenitors (INPs) remains stable, immature granule neurons (GNs) are preserved, and the numbers of mature GNs and glia as well as the DG volume remain unchanged during aging [43]. The QNPs and INPs mentioned above will continue to generate new neurons, a process called adult hippocampus neurogenesis (AHN). Se Hoon et al. found that exercise improves cognition in 5×FAD AD mice by inducing AHN and increasing the level of BDNF [44]. These findings indicate that hippocampal neurogenesis exists in different stages of life, including in healthy older individuals.

During human aging, the decline of brain function is inevitable, but neural tissue has the capacity for plasticity and regeneration, which can resist the physiological and pathological decline in neurodegenerative diseases. Exercise is beneficial to brain function by enhancing hippocampal function [45, 46], increasing neurotrophic factors [47] and improving synaptic plasticity [48].

### Exercise improves brain structure and function

The effect of exercise on the brain structure of the elderly is mainly reflected in the increase of the hippocampal volume. The hippocampus exhibits structural and functional changes in cognitive dysfunction. A meta-analysis including nine studies and 595 AD patients reported that the mean annualized hippocampal atrophy rates were 4.66% (95% CI 3.92, 5.40) for AD subjects and 1.41% (0.52, 2.30) for controls [49]. Recent studies have validated that aerobic and resistance exercise has a positive effect on the improvement of hippocampal volume. In a recent study by Schaeffer et al., 17 PD patients and 18 matched healthy controls were recruited to undertake a 6-week exergaming training program, which combined moderate-intensity aerobic fitness training with elements of coordination and speed. After training, the volumes of CA1, CA4 and DG were significantly increased in the left hippocampus in patients with PD. In particular, the DG region showed the most pronounced effect [50], which is consistent with the results from Frodl et al. [51]. Yun et al. also observed an increase of hippocampal volume after resistance training for 50–80 min a day, three times per week for a total of 24 weeks in elderly women [52]. Moreover, Cariati et al. observed well-organized neurons and glial cells, as well as nerve processes rich in neurotubules and neurofilaments in hippocampal slices of mice after 12 weeks of progressive continuous aerobic exercise [53]. In addition, aerobic exercise can lead to increased functional connectivity of the anterior putamen with the sensorimotor cortex, increased functional connectivity in the right frontoparietal network, and reduced global brain atrophy [54, 55]. A 9-year follow-up of non-demented older adults over 65 years found that walking 72 blocks per week (1600 m/day) leads to volume changes in the perfrontal cortex, parietal cortex, cerebellum, and hippocampus [56].

It is proven that physical exercise can also lead to the improvement of brain function. Alfini et al. found that 12-week treadmill training intervention improved performance in the auditory word learning test, the lexical association test scores, working memory and verbal fluency of MCI patients [57]. Tsai et al. showed that vigorous aerobic exercise for 30 min at moderate intensity, corresponding to 65%–75% of the individual target heart rate reserve (HRR), improved cognitive functions including attention, recognition, decision-making, and memory of elderly adults [58]. A 6-week combined dance and relaxation intervention also enhances motor control and cognition, such as physical balance and attention, in older adults [59]. In older women complaining of memory decline, resistance training twice a week for 6 months improves selective attention, conflict resolution, associative memory and regional patterns of functional brain plasticity [60]. Kujawski et al. recruited healthy older participants in programs of sitting callisthenic balance and resistance training. After the 3-month resistance training program, the participants showed improved decision making, visual attention and global cognitive function [61].

### BDNF: the key modulator between exercise and cognitive function

Neurotrophic factors in the brain may be the core modulator between exercise and cognitive function. BDNF is one of the most widely studied neurotrophic factors in the brain in mammalian health and diseases [62]. It is expressed in the hippocampus and released by glutamatergic neurons and glial cells (such as glial cells isolated from the cortical layer and hippocampus) [63], and is generally thought to be relevant to cognitive function. BDNF can promote axonal sprouting and new branching growth of motor units of the brain [64], enhance the growth of new branches of adjacent surviving neurons and promote generation of key structures around neuromuscular contacts to restore muscle-neuron communication [65, 66]. BDNF may play a critical role in the hippocampal synaptic long-term potentiation (LTP). The LTP is a long-term enhancement of synaptic efficacy thought to underlie learning and memory. BDNF also contributes to neurogenesis and neuronal survival [67]. Converging evidence suggests that deficits in BDNF signaling contribute to the pathogenesis of several major diseases with cognitive disorders, such as AD, Huntington disease (HD), and depression [68].

BDNF is produced in the brain, muscle and hematological system in humans. Current human studies mainly observe the increased level of peripheral BDNF after exercise [69, 70], which may be released from the muscle. In animal studies, increased BDNF content and mRNA have been detected in the hippocampus after exercise. In mice, exercise induces hippocampal BDNF through the proliferator-activated receptor gamma co-activator 1-alpha (PGC-1α)/fibronectin type III domain protein 5 (FNDC5) pathway [71]. Meanwhile, exercise training protects against aging-induced cognitive dysfunction via activation of the hippocampal PGC-1α/FNDC5/BDNF pathway in rats [72].

**Fig. 1:**
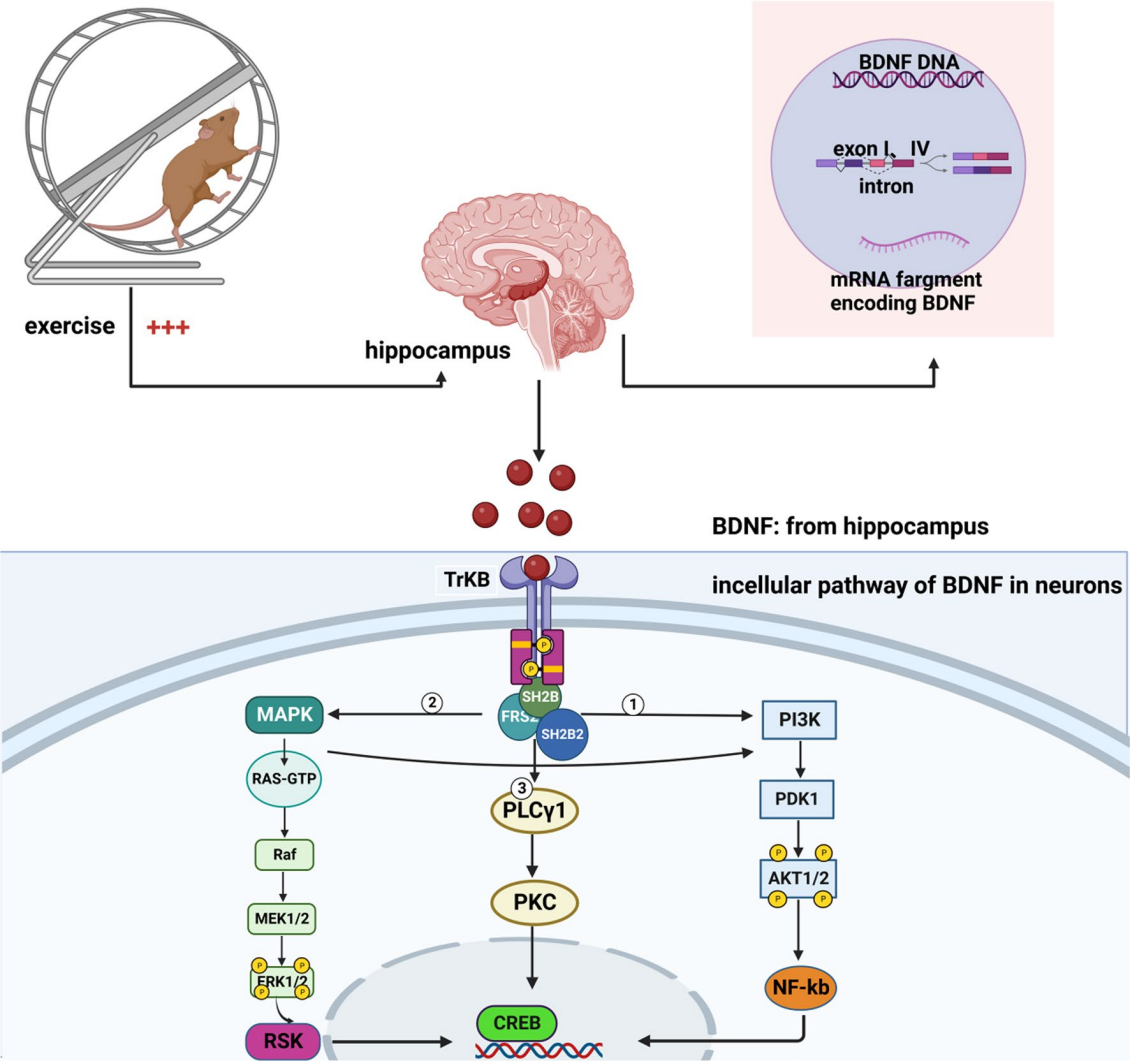
Exercise improves cognitive function by promoting the production of BDNF. During exercise, the amount of BDNF mRNA exons increases, resulting in increased production of BDNF. BDNF enters the brain through the BBB and binds to the TrkB receptor on the cell membrane to activate intracellular signal transduction. ① The BDNF-TrkB complex activates the PI3K/Akt signaling pathway and regulates the survival of neurons. ② The BDNF-TrkB complex activates the MAPK/Ras/ERK signaling pathway and promotes neuronal differentiation. ③ The BDNF-TrkB complex activates the PLC-γ/PKC signaling pathway and regulates synaptic plasticity

BNDF is internalized into neurons mainly by binding to the primary receptor tropomyosin-related kinase B (TrkB), forming the BDNF/TrkB complex. TrkB is a single-channel type I membrane protein that can be incorporated into nuclear endosomes through ligand binding [73]. BDNF binds to TrkB and causes homodimerization and autophosphorylation of TrkB, leading to the recruitment of proteins containing PH and SH2 domains, such as fibroblast growth factor receptor substrate 2, Shc, SH2B and human SH2B adaptor protein 2. The recruited signaling intermediates initiate distinct intracellular signaling cascades and activate three major downstream signaling pathways, including phosphatidylinositide 3-kinase (PI3K), mitogen-activated protein kinase (MAPK) and phospholipase Cγ (PLCγ) intracellular cascades (Fig. 1). Through activation of these intracellular signaling pathways, exercise can resist the process of cognitive disorders in the central nervous system (CNS) [74]. PI3K regulates neuron growth and survival through activation of downstream protein kinase B (Akt) [75, 76]; the PLCγ-mediated pathway regulates synaptic plasticity through the downstream PKC signaling [77]; and the MAPK signaling pathway mainly regulates neuronal differentiation and neurite protrusion [78]. The Rat Sarcoma (RAS) signaling pathway, downstream of MAPK, regulates multiple cellular functions including proliferation. RAS binds to GTP via GDP-GTP exchange factors. RAS activity is also modulated by a complex array of extracellular signal-regulated protein kinase (ERK)-protein-kinase-dependent negative feedback loops [79] (Fig. 1). The negative feedback regulation coordinates the dynamic behavior of the Ras-Raf-MEK-ERK signal transduction pathway. Extensive animal experiments have demonstrated the effects of BDNF/TrkB signaling in improving cognitive function. Xu et al. reported that treadmill exercise could enhance learning and memory ability in healthy male Wistar rats through upregulation of serum BDNF and its receptor TrkB [80]. Timiga et al. found that short-term running exercise increased the levels of exon I and IV mRNA of BDNF in the hippocampus of C57BL/6J mice [81], and single form of exercise increased the level of exon IV mRNA of BDNF in an intensity-dependent manner [82]. Notably, Baranowski et al. demonstrated that exercise elevated BDNF levels in C57BL/6J mice, further reducing the activity of beta-site amyloid precursor protein cleaving enzyme 1 (BACE1), resulting in reduced Aβ production [83]. Therefore, it seems to be the increase of BDNF in the prefrontal cortex and hippocampus and the decrease of BACE1 after exercise that reverse the pathological amyloid deposition to some extent in the AD mice. These results suggest that exercise can act on transcription and translation of BDNF, and thus counter cognitive dysfunction through related pathways.

In addition, several studies have revealed other factors or mechanisms. For example, Martini et al. suggested that power exercise inhibits streptozotocin-induced spatial memory deficits in AD mice by activating the phosphorylation of the hippocampal BDNF/ERK/CaMKII/CREB signaling pathway [84]. Moreover, researchers have proposed that the mechanism by which exercise increases BDNF levels in brain tissue may be due to the release of an endogenous histone deacetylase (HDAC) inhibitor. Exercise induces metabolic changes in the liver, with increased expression of the ketone body β-hydroxybutyrate (DBHB), which is synthesized from acetyl coenzyme A generated by fatty acid β-oxidation and can reach the hippocampus via the circulatory system [85]. DBHB increases the histone acetylase (HAT) and decreases the activity of HDAC in the hippocampus. Therefore, the ratio of HAT to HDAC is increased [86], resulting in reduced binding of HDAC2/3 to the BDNF promoter, which is beneficial to BDNF transcription and expression.

Although physical exercise has been associated with BDNF elevation, the relationship demonstrates a degree of variability. A single-nucleotide polymorphism in the pro-region of the human BDNF gene, which results in a valine (Val)-to-methionine (Met) substitution called BDNF Val66Met, is considered to influence the cognitive function in degenerative diseases [87]. The polymorphism can lead to improper protein folding and reduce the binding of mature BDNF to TrkB, causing impairment in hippocampal function [88]. Meanwhile, the presence of BDNF Val66Met may influence the beneficial effects of exercise, both in humans and in mice [89–91]. For example, physical exercise is able to enhance the hippocampal-dependent memory only in the BDNF Val/Val mice (wild-type), but not in the BDNF Met/Met mice [92]. Canivet et al. observed that physically active Val/Val homozygotes have better episodic memory compared with inactive Val/Val homozygotes, while there is no difference in episodic memory between active Met carriers and inactive Met carriers [93].

### Exercise improves synaptic plasticity

The neuronal synaptic network is the basis for memory formation and retention. Zhu et al. examined the synaptic proteins of excitatory synapses in all brain regions of mice by mapping whole-brain synapses. They found that the higher cognitive functions the brain regions control, the greater synaptic diversity they contain [94]. The synaptic plasticity includes presynaptic plasticity, synaptic cleft plasticity and postsynaptic plasticity, which largely depend on synaptic number and plasticity proteins, such as synaptotagmin and PSD. Synaptotagmin is a presynaptic membrane protein that is widely distributed in presynaptic vesicle membranes. The role of synaptotagmin includes regulation of synaptic vesicle transport and release of neurotransmitter [95]. Meanwhile, postsynaptic mitogens mainly include PSD-95. As a major regulator of synaptic maturation, PSD-95 can interact, stabilize and transport NMDA receptors and AMPA receptors to the postsynaptic membrane [96]. NMDA receptors and AMPA receptors are two major ionotropic receptors to selectively capture neurotransmitters in postsynaptic sites. In synapses between Schaffer collaterals and CA1 pyramidal cells in the hippocampus, exchange of glutamate receptor components, modification of receptors through phosphorylation, and addition of more receptors to the area of the postsynaptic density contribute to LTP [97]. Previous studies found that voluntary exercise or wheel running enhances LTP in the DG of young or healthy rats, probably through increase of synaptic plasticity-related proteins and membrane receptors [98, 99]. This effect of exercise on synaptic plasticity may be more evident in pathological situations. Meanwhile, in healthy individuals or AD patients, accumulation of Aβ alters the conformation of the C-terminal structural domain of NMDA receptor and its interaction with the protein phosphokinase PP1 [100], leading to inhibition of excitatory synapses, LTP and neurotransmission.

Treadmill exercise leads to some morphological changes in 3×Tg-AD mice, including increases in synaptic number, expression of synaptotagmin, axon length, dendritic complexity, and dendritic spine number. Exercise could even restore these parameters to levels in normal control mice [101]. Meanwhile, Li et al. reported that adaptive treadmill training for 12 weeks, 5 days/week, 45 min a day, leads to improved spatial learning and memory in Tg-APP/PS1 mice, with significant increases in the number of synapses in the CA1 region of the hippocampus and in PSD expression. They concluded that exercise alleviates the impairment of postsynaptic plasticity [102]. Another exercise experiment in AD mice showed that aerobic intermittent exercise combining running with swimming for 40 min/day, 6 days/week for 4 weeks, can improve neurogenesis and cognitive behavior, possibly mediated by plasticity proteins such as synaptotagmin and PSD95 [103]. In summary, the exercise-induced morphological and functional changes of synapses in the brain significantly influence the neuronal information exchange network.

## Exercise improves mitochondrial health

Mitochondria serve essential functions in neurons and mitochondrial maintenance directly affects neuronal development, function, and survival. Neurons are highly differentiated cells with a large energy demand, and therefore are highly dependent on mitochondrial function [104]. Mitochondrial damage in the brain will cause bioenergetic deficiency, intracellular calcium dysregulation and oxidative stress, leading to abnormal apoptosis of neurons. Mitochondria produce reactive oxygen species (ROS) in the process of biological oxidation and energy conversion. When there is an imbalance between the generation of ROS and the body’s antioxidant defense system, oxidative stress in mitochondria would cause oxidative stress. Mitochondrial oxidative stress leads to dysregulation of the mitochondrial energy metabolism, which in turn causes damage to mitochondria, thus promoting the development of neurodegenerative disease [105]. Meanwhile, mitochondria play key roles in the development of adult neuroplasticity. For example, during early neuronal differentiation, mitochondria regulate the differentiation and growth of axons by buffering cytosolic Ca2^+^ and thereby promoting polymerization of axonal microtubules [106]. Therefore, maintaining a healthy mitochondrial pool is critical to neuronal health. There are many pathways for mitochondrial quality control, such as the degradation of misfolded mitochondrial proteins, mitochondrial fission and fusion, and the engulfment and degradation of damaged mitochondria termed mitophagy [107]. A distinctive neurobiological feature of neurodegenerative diseases is the presence of excessive oxidative stress and inflammatory responses in the brain [28], while mitochondrial functional impairment is an early pathological hallmark of neurodegenerative disease.

Exercise has been proven to promote mitochondrial biogenesis [108], maintain the balance of neuronal mitochondria, reduce excessive oxidative stress caused by mitochondrial damage, and improve ATP production [109]. Meanwhile, exercise can improve mitochondrial health in the brain by modulating mitochondrial biogenesis, kinetics (fusion/fission) and autophagy.

### Mitochondrial biogenesis

**Fig. 2:**
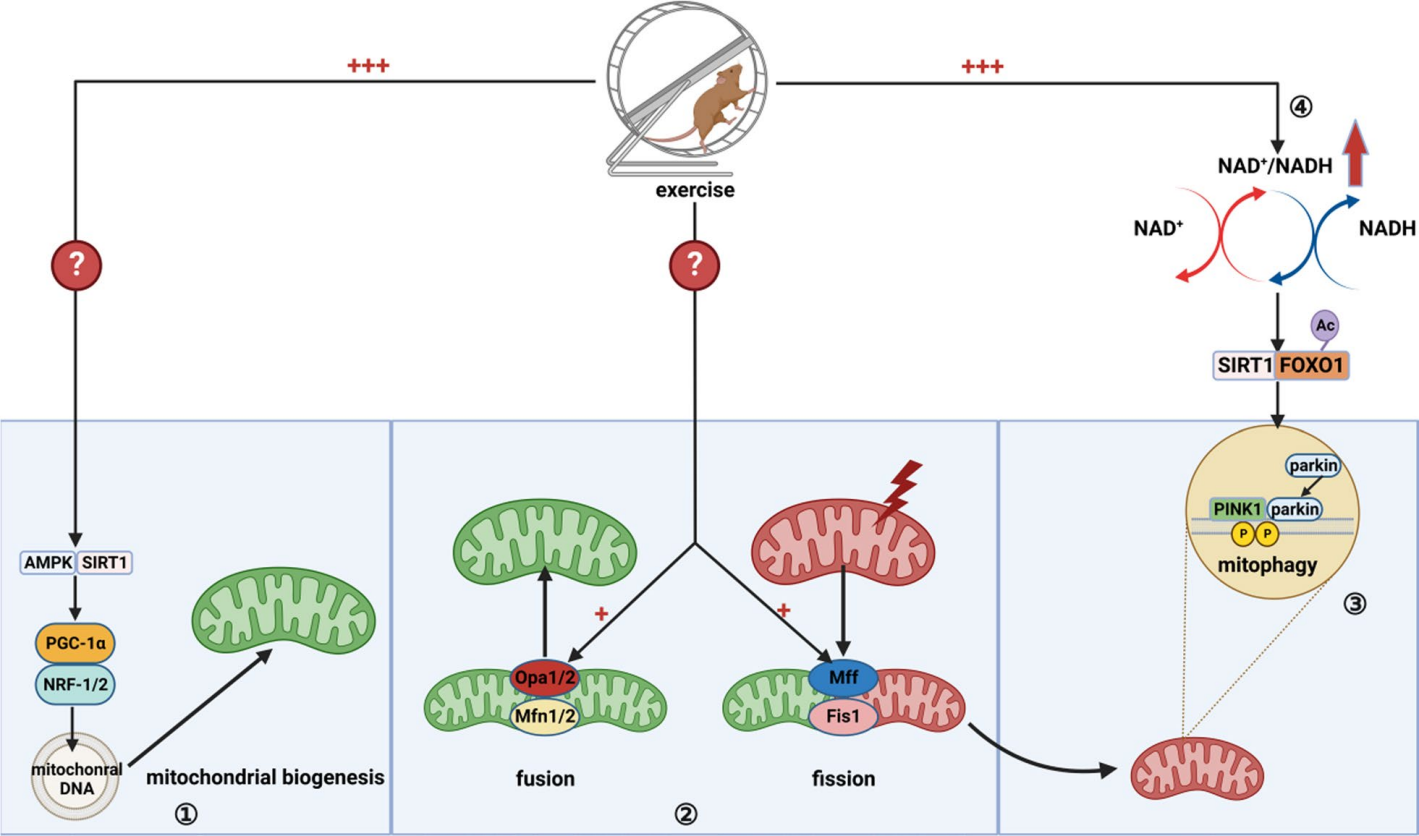
Exercise benefits the stability of neuronal mitochondria and improves the oxidative stress and energy supply. ① Mitochondrial biogenesis: exercise can promote the AMPK/SIRT1/ PGC-1α pathway, recruit more NRF1/2 and then upregulate the mitochondrial gene expression. ② Fusion and fission: exercise can improve the fusion and fission proteins in mitochondria, such as Opa1/2, Mfn1/2, Mff, and Fis1. These proteins are especially important for the “metabolism” of mitochondria. ③ Mitophagy: exercise can promote the PINK/parkin pathway of mitophagy in the outer mitochondrial membrane (OMM). ④ After exercise, more NADH is converted to NADH+, resulting in the acetylation of FOXO1. This urges PINK1 to recruit more Parkin in the OMM to remove damaged mitochondria

Studies have demonstrated that exercise promotes mitochondrial biogenesis by inducing expression of transcription coactivator peroxisome PGC-1α, MAPK and Sirtuin 1 (SIRT1) [75, 76]. PGC-1α is a transcription coactivator involved in mitochondrial biogenesis and regulation of antioxidant defense [77]. Once activated through phosphorylation or de-acetylation, PGC-1α activates nuclear respiratory factor 1/2 (NRF1/2) and subsequently mitochondrial transcription factor A (Tfam) [110]. Activation of the PGC-1α–NRF–Tfam pathway leads to synthesis of mitochondrial DNA and proteins, and generation of new mitochondria. MAPK is an intracellular energy status sensor that maintains energy stores by regulating anabolic and catabolic pathways [78]. MAPK is activated during both exercise and muscle contraction in vivo, and is considered to be an important signaling molecule in the regulation of muscle metabolism during exercise and adaptation of skeletal muscle to exercise training [111]. SIRT1 may activate energy metabolism, neurogenesis and mitochondrial quality control through regulation of multiple transcription factors, including PGC-1α. An animal study showed that 8 weeks of treadmill exercise elevated the expression of SIRT1 and PGC-1α and reduced α-synuclein deposition levels in PD mice [112]. The exercise also increased levels of biomarkers of mitochondrial biogenesis, antioxidant enzymes, as well as key factors of mitochondrial autophagy. These results suggest that chronic aerobic exercise may increase mitochondrial biogenesis and autophagic response through SIRT1/PGC-1α to reduce abnormal protein deposition in neurodegenerative diseases, thus improving cognitive function (Fig. 2).

### Mitochondrial kinetics

Mitochondrial fusion is mainly mediated by pro-fusion proteins optical atrophy 1/2 (Opa1/2) and mitochondrial fusion protein 1/2 (mitofusin1/2, Mfn1/2), which expand the area of mitochondrial network by facilitating the fusion of the inner and outer mitochondrial membranes (OMM). Mitochondrial fragmentation is mediated by mitochondrial fission factor and fission protein 1, which recruit dynamin-related protein 1 (Drp1) to the OMM, separating dysfunctional organelles from the mitochondrial network and allowing the isolated mitochondrial fragments to be degraded by autophagic lysosomes [30, 108]. Marques-Aleixo et al. reported that PD mice with treadmill endurance training or having free-wheel voluntary physical activity have increased expression of the fusion proteins Mfn1/2 and Opa1, and decreased expression of the cleavage protein Drp1, as measured by Western blotting [113]. These results suggest that the exercise-induced favorable alterations in mitochondrial dynamics and autophagic signaling may contribute to increased mitochondrial plasticity (Fig. 2).

### Mitochondrial autophagy

Mitochondrial dysfunction causes deficient energy production, oxidative stress and impaired cell signaling, all of which are associated with the pathogenesis of cognitive dysfunction. Clearance of dysfunctional mitochondria is important for maintaining normal neuronal function [114, 115]. Following mitochondrial damage, autophagy is induced in three ways: ubiquitin-mediated mitochondrial phagocytosis, including PTEN-induced putative kinase 1 (PINK1)–Parkin (an E3 ubiquitin ligase)-dependent phagocytosis [116]; OMM receptor-mediated mitochondrial phagocytosis [117]; and lipid-mediated mitochondrial phagocytosis [118]. Exercise exerts beneficial effects in cognitive impairment diseases mainly through the first pathway. In the PINK1–Parkin pathway, PINK1 first accumulates on the OMM, which is induced by the loss of membrane potential in damaged mitochondria. The stabilized PINK1 recruits Parkin to the membrane, and then the PINK1/Parkin ubiquitinates the substrates on OMM to activate the autophagic machinery (Fig. 2) [119, 120]. A mediator of exercise that can activate the PINK1/Parkin pathway may be the nicotinamide adenine dinucleotide (NAD) [121, 122]. NAD^+^ is an important cofactor for SIRT [33]. Exercise significantly increases the intracellular NAD^+^/NADH ratio, leading to increased expression of SIRT1 in the brain [123]. The NAD^+^–SIRT1 signaling acts on mitochondrial forkhead box O (FOXO) [124], mediating FOXO1 deacetylation [125], and then activates the PINK1–Parkin pathway [126], leading to mitochondrial fission and activation of mitochondrial autophagy (Fig. 2). Furthermore, the effect of exercise in increasing NAD levels may be partly mediated through a cyclic GMP-AMP synthase (cGAS)–interferon gene stimulating hormone (STING)-dependent pathway. Hou et al. found that in APP/PS1 mutant transgenic mice, increasing NAD levels could reduce DNA damage. The underlying mechanisms may include decreased expression of inflammatory cytokines in the brain, decreased abnormal activation of microglia and astrocytes, and decreased activation of the CGAS–STING pathway [127].

## Exercise improves cognitive function by promoting cytokine release

**Fig. 3:**
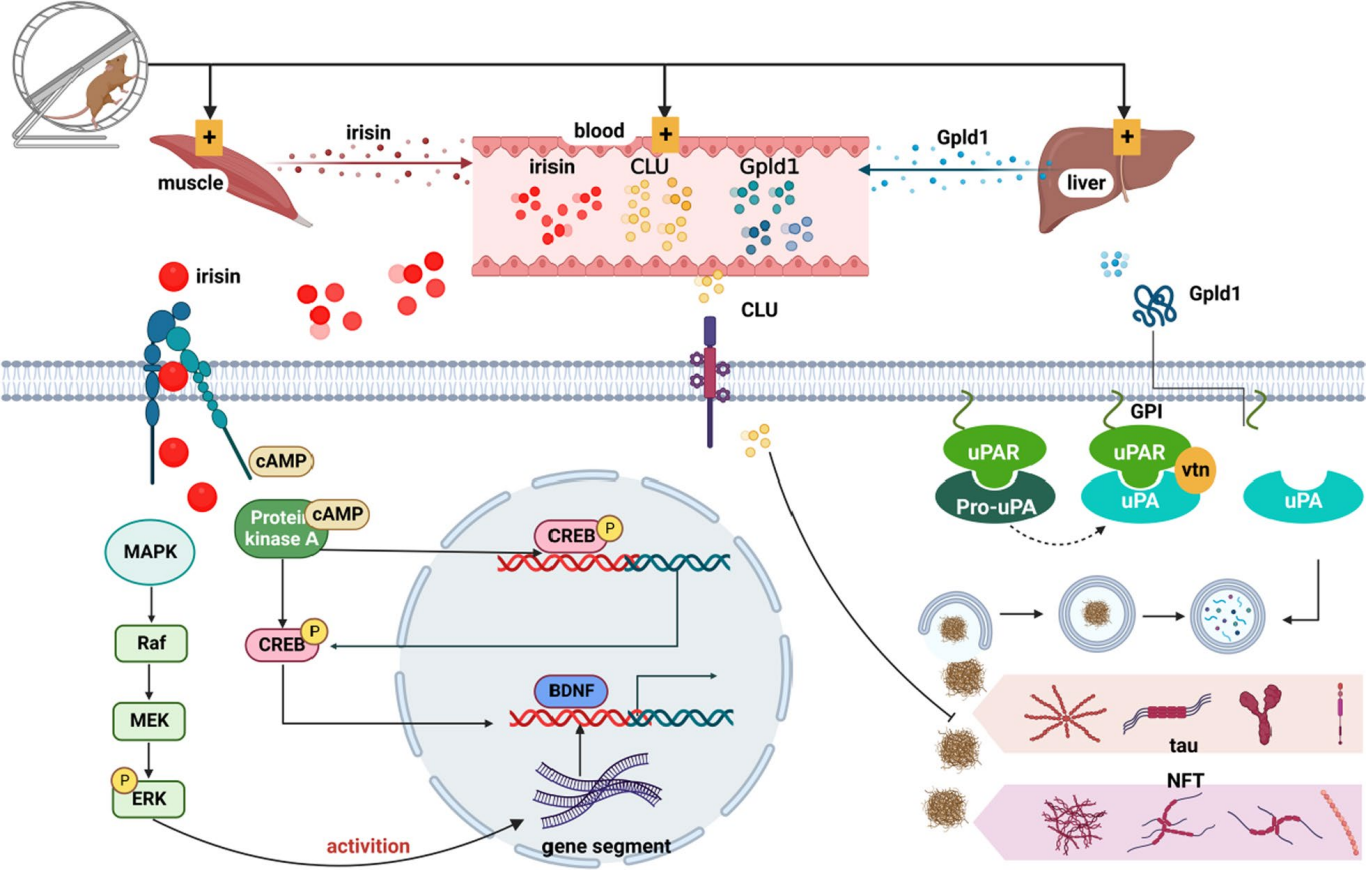
Exercise can stimulate various organs in the body to secrete corresponding cytokines. Muscle: after exercise, skeletal muscle secretes a large amount of Irisin into the peripheral system. Irisin is internalized into cells by binding to integrin receptors and passes through the blood–brain barrier, activating the MAPK/ERK and cAMP/CREB pathways and promoting the production of BDNF. Liver: exercise stimulates the liver to secrete Gpld1 into the blood, a specific phospholipase that can reduce amyloid precipitation anchored by GPI on the neuronal cell membrane. Blood: exercise enhances the level of clusterin (CLU). LRP8, a special receptor in the brain, mediates the entry of CLU into the cytoplasm. CLU can inhibit Aβ aggregation and neurofibrillary tangles

How does skeletal muscle come in contact with the brain? Currently, skeletal muscle is considered to be a secretory organ of the body that releases various cytokines into the circulatory system to communicate with other organs of the body [128]. Physical exercise promotes the secretion of cytokines and hormonal molecules from peripheral tissues and organs, including skeletal muscle, to act on neurons in the brain after crossing the brain–blood barrier (BBB). Then they promote the secretion of neurotrophic factors [129], reduce immune inflammatory responses, relieve stress on brain tissue, and improve synaptic plasticity and protective effects on neurons [130]. These factors mainly include irisin [131–133], clusterin (CLU) [134], and glycosylphosphatidylinositol-specific phospholipase D1 (Gpld1, a liver-derived factor) [112, 135] (Fig. 3). They may ameliorate cognitive dysfunction through all of the above processes.

### The key factor of skeletal muscle-brain axis: irisin

Irisin is released into the circulatory system by skeletal muscle during exercise. It is released on cleavage of the membrane-binding precursor protein FNDC5, which stimulates browning and thermogenesis of adipocytes [136]. Irisin is an important modulator of cognitive function under exercise therapy [137] and has the highest affinity for av/β5 integrin in skeletal muscle [138]. FNDC5/irisin is highly sensitive to exercise stimuli. Recent studies have identified that exercise may improve cognitive function through FNDC5/Irisin [139, 140]. For example, boosting brain FNDC5/irisin levels can rescue synaptic plasticity and memory defects in a mouse model of AD [141]. Islam et al. found that genetic deletion of FDNC5/irisin (global FNDC5 knock-out (KO) mice; F5KO) impairs cognitive function in exercise, aging and AD [137]. The diminished pattern separation in F5KO mice can be rescued by delivering Irisin directly into the DG. In addition, the F5KO mice receiving voluntary free-wheel exercise fails to show exercise-induced improvements in spatial learning and memory in comparison to the WT mice with the same amount of exercise. Furthermore, in F5KO mice, adult-born neurons in the DG showed abnormal morphology, transcription, and function [137].

Irisin reaches the CNS through the BBB after exercise and activates the MAPK/ERK and cAMP/protein kinase A (PKA)/CREB signaling pathways, resulting in increased expression of BDNF in neurons (Fig. 3). Li et al. found that irisin treatment increases the levels of phosphorylated Akt and ERK1/2 in middle cerebral artery occlusion mice. When ERK1/2 expression was blocked by specific inhibitors, the neuroprotective effect of irisin disappeared. These results suggest that irisin may exert its neuroprotective effect through the MAPK/ERK signaling pathway (Fig. 3) [142]. Phosphorylation of MAPK/ERK activates the induction of a large number of activity-regulated genes related to cognition in the nucleus, such as Arc/Arg3.1, Arl5b, Gadd45b, Homer1, Inhba, and Zwint [143]. Activity-dependent gene expression is important for the formation and maturation of neuronal networks, neuronal survival, and plastic modifications within mature networks [144]. The phosphorylation of MAPK/ERK initiates the transcriptional process in the hippocampus (Fig. 3) [145]. Sun et al. performed treadmill training on AD model mice injected with Aβ in the hippocampus, and found that physical exercise prevented Aβ-induced cognitive deficits in object recognition tasks and Morris water maze. Further studies showed that exercise regulated neurogenesis and brain immune activity by altering ERK modifications and controlling MAPK/ERK signaling pathways [146].

A recent study found that recombinant Irisin stimulates the cAMP/PKA/CREB pathway in cortical slices of AD mice [141]. This pathway has been shown to inhibit the transcription and expression of endogenous tau protein, which is thought to be involved in the molecular mechanism regulating cognitive dysfunction, modulating neuroplasticity, and preventing memory dysfunction [147]. The Irisin-induced CREB phosphorylation can be abolished after application of PKA inhibitor, myristoylated PKI 14–22 [141]. Exercise can promote peripheral cAMP and CREB levels in mice. As an intracellular second messenger, cAMP undergoes conformational change upon binding to the regulatory subunit of PKA, leading to release of the catalytic subunit and phosphorylation of the substrate of cAMP, thereby promoting the release of neurotransmitters from synaptic connections between neurons. In contrast, CREB, the DNA transcription-binding factor of BDNF, can promote neurogenesis by increasing the activity of RNA polymerase of BDNF to regulate the expression of BDNF gene in the hippocampus (Fig. 3) [148]. Lee et al. reported that short-term memory of cerebral ischemic mice was improved after treadmill exercise. Further molecular evidence showed increased phosphorylation of the Akt-CREB-BDNF pathway and decreased neuronal apoptosis in the hippocampus [149]. Ko et al. found that treadmill training for 8 weeks inhibited phosphorylation of CREB in the hippocampus and increased the CREN/p-CREB ratio in mice with traumatic brain injury (TBI). These results suggest that treadmill exercise improves spatial learning by improving the CREB/BDNF/TrkB signaling pathway in TBI mice [150].

### Other cytokines in the skeletal muscle-brain axis

Recently, more and more cytokines are being found in the crosstalk between skeletal muscle and brain. Cathepsin B (CTSB), a muscle secretory factor, is important for the cognitive and neurogenic benefits of running. Moon et al. have found that running increases CTSB levels in mouse gastrocnemius muscle and plasma. Furthermore, application of recombinant CTSB enhances the expression of BDNF and doublecortin (DCX) in adult hippocampal progenitor cells through a mechanism dependent on the multifunctional protein P11. The findings suggest that CTSB is a mediator of effects of exercise on cognition in the skeletal muscle-brain axis [151]. Exercise-induced apelin is demonstrated to be beneficial to sarcopenia. Recently, evidence shows that apelin-13, an active form of apelin, suppresses neuroinflammation and improves cognitive decline in diverse pathological processes. An animal study found that apelin-13 may upregulate the glucocorticoid receptor level and nuclear translocation in hippocampus [152]. Moreover, kynurenine accumulation can be suppressed by activating kynurenine clearance in skeletal muscle after exercise, benefiting the brain. Cervenka et al. have discussed peripheral mechanisms of tryptophan-kynurenine metabolism and their effects on inflammatory, metabolic, and psychiatric disorders [153].

### CLU

Physical exercise is generally beneficial against cognition decline, which is associated with reduced inflammation in the hippocampus. Yet, little is known about the mechanisms. CLU, also known as apolipoprotein J, is a multifunctional glycoprotein present in plasma [154]. CLU expression is near-ubiquitous, and CLU mRNA is found in almost all cell types. CLU expression is increased in response to a broad variety of signals and conditions, including the presence of oncogenes, numerous growth factors and cytokines, as well as many stress- or apoptosis-inducing conditions/agents such as heat shock, UVA, UVB, proteotoxic stress, heavy metals, oxidants, hypoxia, ionizing radiation, and chemotherapeutic drugs [44]. Recently, a study found that injecting the ‘exercise plasma’ collected from voluntarily running mice into the sedentary mice induces reductions in baseline expression of neuroinflammatory genes and experimentally induced brain inflammation [155]. The authors also performed a proteomic analysis of the plasma composition in exercising mice and found that the levels of specific cytokines, such as CLU, glycoprotein pigment epithelium-derived factor (PEDF), and leukemia inhibitory factor receptor (LIFR), are increased after exercise [155]. When mice were further injected with exercise plasma without CLU, PEDF, or LIFR, the gene expression and protein levels of anti-inflammatory factors were significantly reduced in the CLU-deficient mice. Moreover, the researchers found increased plasma CLU levels as well as improved cognition and memory in patients with MCI after 6 months of physical activity intervention [155]. LRP8, also known as APOER2, is the receptor of CLU [156] and expressed most highly in brain endothelial cells and neurons [155]. This indicates that elevated CLU in blood may bind to brain endothelial cells through LRP8 receptors and benefit the brain. Protein aggregation is a common pathological hallmark across neurodegenerative diseases. Similar to CLU co-localizing with Aβ in the senile plaques, in the case of α-synucleinopathies, CLU co-localizes with cortical Lewy bodies in the brain. Moreover, the co-expression of CLU may protect neurons from proteotoxicity [157]. In mice injected intravenously with recombinant clusterin (rCLU) labelled with Atto-647 N fluorophore, researchers found a prominent decoration of the cerebrovasculature of the hippocampus with Atto-647 N-rCLU [155], which demonstrated that endothelial cells and neurons may be the immediate target of circulatory CLU.

Besides anti-neuroinflammation, researchers have also summarized the effects of CLU on AD, including Aβ aggregation and clearance [158], neurofibrillary tangle and tau protein phosphorylation [159]. de Retana et al. treated 14-month-old APP23 transgenic mice with subchronic intravenous CLU for 1 month and measured the concentration and distribution of Aβ in the brain, as well as the levels of Aβ in plasma and cerebrospinal fluid after treatment. The results showed that CLU treatment prevented the accumulation of Aβ in cerebral arteries and induced a reduction of total brain insoluble Aβ [160]. Wojtas et al. found that CLU has a significant effect on anxiety-like behavior in the context of tau pathology, as manifested in the open field test and elevated plus maze [159]. In vitro studies found that CLU significantly inhibits tau protein fibrillation and neurofibrillary tangle formation. Down-regulation of CLU leads to increased α-synuclein aggregation in SH-SY5Y cells stably overexpressing α-synuclein [161], which may exacerbate the course of cognitive impairment of PD. These studies illustrate the beneficial role of CLU in improving cognitive dysfunction during ageing or in neurodegenerative diseases. Therefore, CLU may be one of the important mediators of the effects of exercise on the CNS.

### The liver-brain axis: Gpld1

Gpld1 is a phospholipase that can cleave important proteins fixed to the cell membrane via glycosylphosphatidylinositol (GPI). The lysed proteins are released from the cell surface to perform different biological functions (Fig. 3) [162]. Previous studies have found that Gpld1 plays a beneficial role in the pathological processes of chronic diseases, such as chronic liver disease, diabetes and cancer, by cleaving proteins such as triglycerides [163], proteoglycans [164], and carcinoembryonic antigen [165].

In a recent animal study by Horowitz et al., researchers found that systematic blood plasma administration can transfer the benefits of exercise to the aged hippocampus. The levels of BNDF and neurogenesis increase in the hippocampus, and the number of mature neurons expressing 5-bromo-2′-deoxyuridine and Dcx increases significantly. Furthermore, the ability of mice to perform radial arm water maze (RAWM) and novel object recognition tasks is also improved, suggesting improvement of the cognitive ability of elderly mice [112]. The authors also sought to identify individual circulating blood factors that mediate these effects. They used isobaric tagging together with liquid chromatography-tandem mass spectrometry to measure relative amounts of soluble proteins in the plasma, and found that 63% and 67% of exercise-induced factors in aged and mature mice, respectively, are predominantly expressed in the liver, in which Gpld1 and paraoxonase 1 are overrepresented [112]. The increased Gpld1 concentrations in plasma are correlated with improved cognitive performance in the RAWM and contextual fear conditioning behavioral test in aged mice [112].

Mechanistic studies suggest that exercise may improve age-related regeneration and cognitive impairment by altering the expression of related proteins in the urokinase-type plasminogen activator receptor (uPAR) signaling pathway downstream of GPI anchored substrate cleavage (41 coagulation and complement proteins in the uPAR signaling pathway were found to be reduced after exercise) [112]. The study suggested that Glpd1 may be one of the key cytokines acting in the muscle-brain axis, but more in-depth studies are still needed to validate the underlying molecular mechanism.

In addition to the cytokines mentioned above, recent studies have also found that the proteasomal adaptive response induced by skeletal muscle stress is also important for protein quality control in the brain, reducing age-related proteasomal substrate accumulation [166]. NADPH oxidase also protects against ageing and oxidative stress in the brain. A recent study found a biological mechanism by which liver metabolites regulate synaptic activity in the prefrontal cortex by enhancing synaptic related RNA methylation in the brain after exercise, thus preventing the occurrence of anxiety-like phenotypes [167].

The link between exercise and specific cytokines is gradually being explored and validated. In summary, exercise can act as a stimulator for body organs, including skeletal muscle, allowing secretion of specific factors that act on brain tissue.

## Exercise improves cognitive function by improving brain metabolism

Glucose is the main energy supplier in the brain, while ketone bodies and lactate provide a small proportion of energy. Healthy energy metabolism is beneficial to cognitive function. Glucose plays an important role in a variety of neurological processes such as the synthesis and recycling of neurotransmitters, and influences processes of message transmission and synaptic remodeling [33]. Dysfunctional glucose metabolism is present in brain regions of patients with neurodegenerative diseases. For example, in the hippocampus, hypothalamus, striatum and insular cortex of patients with AD, the glucose metabolism is reduced [168]. Therefore, these regions are considered to be characteristic areas of AD pathogenesis [168].

### Glucose metabolism

**Fig. 4:**
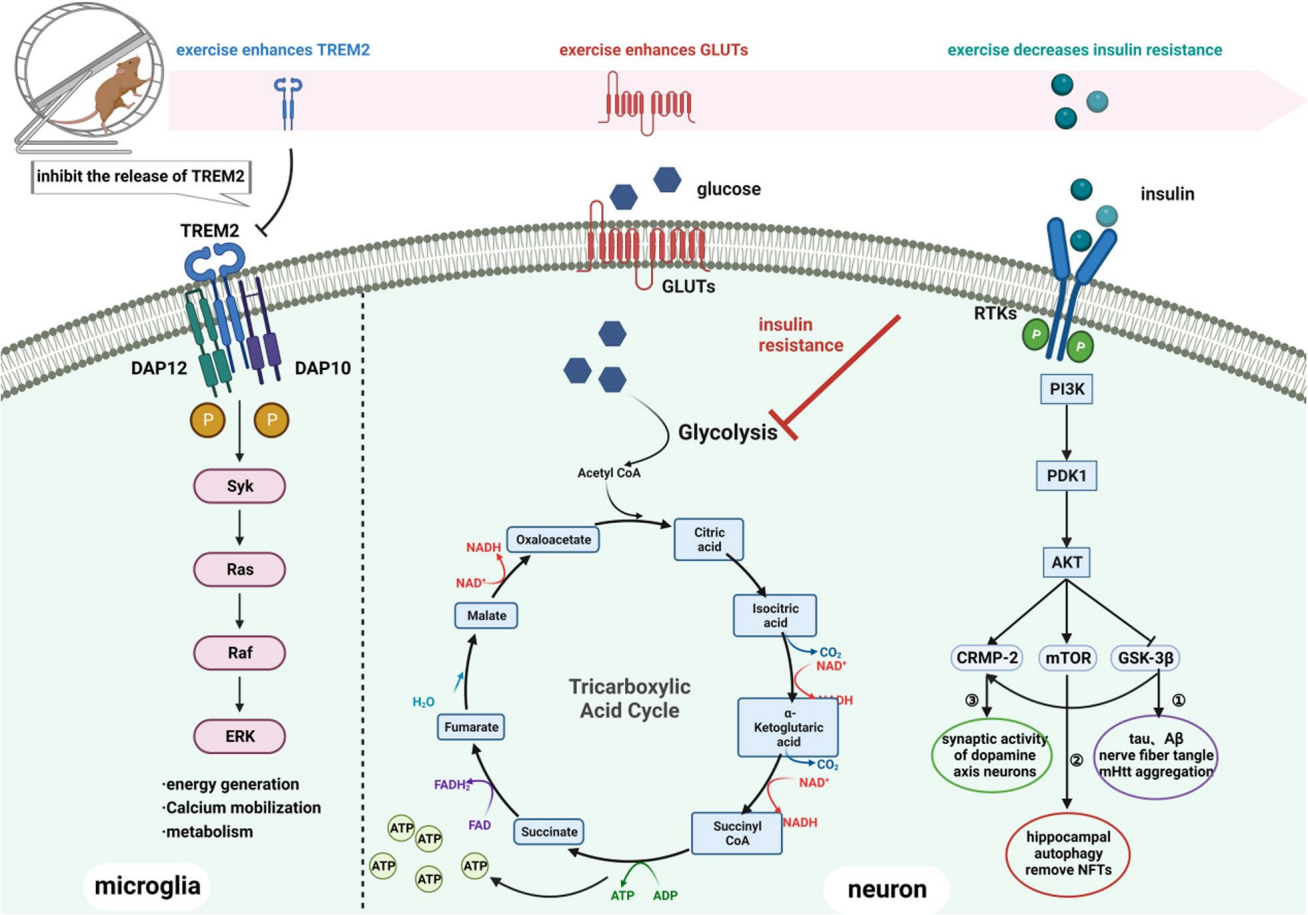
Exercise can improve the energy metabolism of the brain, which is beneficial to cognitive function. Insulin resistance: exercise reduces insulin resistance in the brain, allowing more insulin to reach the brain through the blood–brain barrier. Activation of the PI3K/Akt pathway can lead to activation of mTOR and CRMP-2 as well as inhibition of GSK3β, resulting in ① decreased deposition of Tau and Aβ proteins, neurofibrillary tangles (NFTs), and aggregation of mHtt; ② activation of hippocampal autophagy and clearance of NFTs; and ③ improved synaptic activity of dopaminergic neurons. Glucose metabolism: exercise increases the amount of GLUTs that can remove more glucose to the cytoplasm. Glucose undergoes glycolysis and the tricarboxylic acid cycle to provide more ATP to brain tissue. Microglia: TREM2, by binding to DAP10/12, promotes Syk phosphorylation and regulates metabolic processes, including energy generation, calcium mobilization and brain metabolism

Glucose utilization in brain tissue mainly includes glucose transport and intracellular glucose catabolism. It has been hypothesized that various pathogenic cascades caused by impaired glucose metabolism in brain may lead to neuronal degeneration, thus leading to cognitive dysfunction [169]. Glucose is transported to various types of brain cells mainly through glucose transporters (GLUTs), of which GLUT1 is mainly distributed in the BBB endothelial cells and GLUT3 is concentrated in neuronal axons and dendrites. Both are important pathways that mediate neuronal glucose uptake (Fig. 4) [170]. A recent study showed that regular exercise enhances the expression of GLUT1 and GLUT3 in the CNS and improves spatial learning and exploration as assessed by the Morris water maze test in AD model mice [171]. Similarly, exercise also improves glucose metabolism by inhibiting HDAC4 and upregulating GLUT1 expression. This effect may be achieved by increasing glycolysis, respiratory function, and ATP production (Fig. 4) [172].

### Insulin resistance

Insulin resistance is often present in brains of CNS disorders, resulting in an energy deficit in the brain that suffers from oxidative and metabolic damage [173]. A recent study showed that exercise promotes insulin to cross the BBB into brain tissue and improves its binding to blood vessels in the mouse brain [174]. Insulin/PI3k/Akt is an important signaling pathway for insulin transmission in the brain, maintaining glucose homeostasis and metabolism [175]. Exercise can inhibit downstream activation of glycogen synthase kinase-3β (GSK-3β) by reducing insulin resistance in the brain and activating the PI3K/Akt pathway [146, 176]. GSK-3β is a key factor causing abnormal protein aggregation and NFTs in the brain, which can lead to the disruption of neuronal function and synaptic damage [177]. The presence of excess GSK-3β in brain tissue can cause irreversible impairment of cognitive function. GSK-3 is now considered as an early biomarker of AD (Fig. 4) [178]. In addition, in HD, neurotoxicity is attenuated by GSK-3β inhibitors. Inhibition of GSK-3β activates mitochondrial lysosomes, leading to timely clearance of toxic aggregates of mutant Huntingtin [179]. In PD, collapsed response mediator protein 2 (CRMP-2), a downstream signal factor of PI3K/Akt/GSK-3β, regulates the degeneration of axons and dendrites of dopaminergic neurons through microassembly [180]. It may also be one of the possible mechanisms by which exercise improves PD cognitive function. Studies have demonstrated that swimming exercise activates PI3K/Akt, resulting in reduced phosphorylation of GSK-3β and increased phosphorylation of CRMP-2, reducing the vulnerability to stress and mediating neuroplasticity in mice (Fig. 4) [146].

As a physiological stimulus, physical exercise can also promote glucose uptake and energy metabolism through activation of AMPK [181] and mammalian target of rapamycin (mTOR) (Fig. 4) [182]. Zheng et al. found that the PI3K/Akt/mTOR signaling pathway plays a role in regulating hippocampal autophagy and removing NFTs [183]. Meanwhile, Kang et al. found that treadmill exercise produces beneficial effects on PI3K/Akt/mTOR, autophagy and tau protein hyperphosphorylation in the cerebral cortex of NSE/htau23 transgenic mice [182]. Both experiments eventually observed improvements of cognitive function in mice.

### Microglia

Glucose metabolism in microglia is also important for maintaining normal cognitive function of the brain [184]. Piers et al. found significant metabolic abnormalities in the microglia of AD patients with myeloid 2 (TREM2) gene variants, which included a failure of the glycolytic immunometabolic switch [185]. Microglia also play a key role in the elimination of abnormal protein aggregation in the brain in neurodegenerative diseases [186]. TREM2 is a cell-surface transmembrane glycoprotein that is highly expressed in microglia [187]. It can mediate intracellular signaling by binding to the DNAX activating protein 12/10, and plays a role in promoting SYK (Tyr525/526) phosphorylation, regulating calcium mobilization, mTOR signaling pathway, AMPK signaling pathway, etc. (Fig. 4) [188]. TREM2 is also necessary for microglia-related regulation of synaptic plasticity and participates in the communication between microglia and other cells. AD mice with knockout of *Trem2* at late stage (6–10 months) show accelerated synaptic dysfunction because of more severe amyloid deposition caused by depression of microglia phagocytosis [189]. Evidence shows that TREM2 responds to extracellular signals, such as IL-1β, TNF-α, ROS, and even solute TREM2 (sTREM2), and increases the secreta of microglia, that regulate synaptic plasticity [190].

Recent studies believe that microglia are involved in the improvement of synaptic plasticity through the TREM2 pathway after running exercise. In APP/PS1 AD mice, microglial number, protrusion length and endpoints are increased after long-term voluntary exercise, together with increased protein levels of GLUT5, TREM2, and phosphorylated SYK. The authors found more TREM2-positive fluorescent labels in the microglia of AD-Running mice. In addition, the levels of TREM2 in the hippocampus are significantly increased, while sTREM2 in the plasma is significantly reduced in AD-Running mice compared with AD-Sedentary mice. These results indicate that running exercise upregulates the TREM2 protein in the hippocampus, reduces TREM2 shedding and prevents sTREM2 release into the blood in APP/PS1 mice [191]. In summary, inhibiting the loss of TREM2 may be one of the mechanisms by which running exercise improves glucose metabolism in the hippocampus and rescues cognitive decline in APP/PS1 mice.

## Exercise improves cognition through regulation of gut microflora

Neurodegenerative diseases are often accompanied by dysbiosis of the gut flora [136] and disruption of intestinal barrier [192]. An unhealthy gut can cause abnormal neuroinflammation and oxidative stress. Some products of intestinal flora are also involved in the pathology of abnormal protein aggregation in cognitive dysfunction [193, 194].

### The modulator between gut and brain: *Akkermansia muciniphila*

*Akkermansia muciniphila* (AKK) is considered to be a genus of bacteria that produces beneficial effects in neurodegenerative diseases. It is observed that the abundance of AKK decreases during the pathological process of cognitive disorders. EranBlacher et al. injected 11 different symbiotic bacteria into amyotrophic lateral sclerosis mice to explore their association with the severity of cognitive impairment. They found that cognitive function is improved in mice injected with AKK [195]. Meanwhile, Laura et al. observed a compensatory increase in AKK during the early course of progressive MS, which shows a negative correlation with the symptoms of disability [196]. Ou et al. demonstrated the protective role of AKK against cognitive deficits and amyloid pathology in a mouse model of AD. The ameliorative effect of AKK treatment on cognitive function can be attributed to the repair of metabolic disorders and, concomitantly, reduction of Aβ pathology [197].

In a recent study, APP/PS1 AD model mice and WT mice performed treadmill exercise for 45 min per day, 5 days a week, for 8 weeks with gradual increase of load period and 4 weeks with a constant load period. The results showed that treadmill exercise enriched the intestinal microbial community composition in AD-exercise mice compared with AD-sedentary [198]. Furthermore, the dominant species in the AD-exercise group were *Bacteria, Verrucomicrobiota, Verrucomicrobiae, Verrucomicrobiales, Akkermansiaceae*, and *Akkermansia*. In the review of the previously reported effect of AKK on intestinal mucosal [199, 200], the authors speculate that treadmill exercise can protect the intestinal barrier and provide beneficial effects on the brain by increasing AKK abundance [198].

### The intestinal barrier

**Fig. 5:**
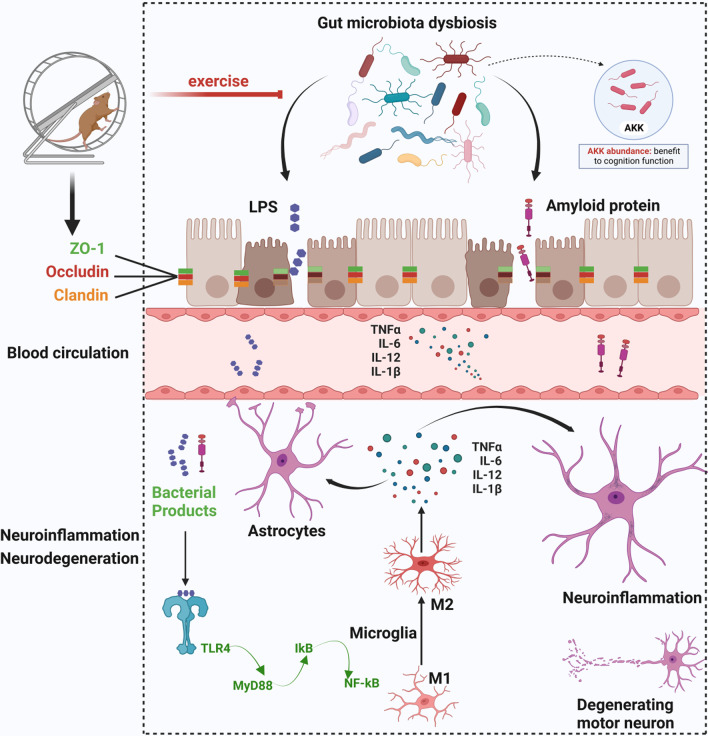
Exercise improves cognitive function through the gut-brain axis. In the context of disrupted intestinal barrier, LPS and amyloid proteins may enter the brain. LPS activates aberrant polarization of astrocytes via TRL4/MyD88/lkB/NF-kB and produces large amounts of pro-inflammatory factors. This is immediately followed by microglia-mediated neuroinflammation and degeneration of motor neurons, ultimately leading to cognitive dysfunction. Exercise improves the integrity of the intestinal epithelium and increases the levels of tight junction proteins, such as ZO-1, Occludin, and Claudin. A stronger intestinal barrier reduces the entry of LPS and amyloid into the brain, and attenuates the above reactions within the brain

Neurodegenerative diseases are often accompanied by inhibition of tight junction protein gene expression and decreased levels of tight junction proteins ZO-1, Occludin, and Claudin. The intestinal epithelium is damaged, microvilli are sparse, and paracellular space is widened, resulting in increased intestinal permeability. When the intestinal barrier and BBB are disrupted, products of the intestinal flora, such as lipopolysaccharide (LPS) [201] and amyloid [202], would leak through the barrier into brain tissue, leading to activation of inflammatory response, neuronal apoptosis and abnormal protein deposition in the brain (Fig. 5). Studies have confirmed that LPS in the brain will activate the Toll-like receptor 4 (TLR4)/myeloid differentiation primary response 88 (MyD88)/nuclear factor kappaB (NF-kB) signaling pathway in neurons [203], causing elevated expression of MyD88, p-IkB-α and NF-kB and decreased expression of IkB-α (Fig. 5). As a transcription factor downstream of the TLR4/MyD88 pathway, NF-kB can stimulate the change of microglia from phenotype M1 to M2. M2 microglia, also known as amoeboid microglia, have an anti-inflammatory and neuroprotective function, which produce anti-inflammatory cytokines (TNF-α, IL-1β, IL-6) and thereby activate the expression of iNOS and COX2 (Fig. 5). Ning et al. used intraperitoneal injection of LPS to observe the induction of neuroinflammation and apoptosis in mice. The results showed that the levels of TNF-α, IL-1β, MDA and ROS in brain tissue increased 8 h after administration, and the numbers of neurons in CA1, CA3, and cerebral cortex were decreased (Fig. 5) [204].

Physical exercise exerts neuroprotective effects probably by maintaining a normal intestinal mucosa and intestinal barrier. Campbell and colleagues reported that 12 weeks of voluntary wheel running improves intestinal epithelial membrane integrity, reduces intestinal inflammation, and increases microbial diversity in mice [201]. In a recent study, Shin et al. found that 4 weeks of treadmill exercise (12 m/min for 30 min/day in week one, with an increase of 2 m/min every week) results in increased expression of the tight junction proteins claudin-1 and occludin, and decreased serum level of LPS in C57BL/6J mice, as detected by Western blotting. The result suggests that plate exercise can increase the expression of tight junction proteins in aged mice, protect the permeability of the intestinal barrier and maintain a stable intestinal environment [205]. Therefore, it can be concluded that exercise plays a role in maintaining both intestinal flora and intestinal barrier stability and has beneficial effects on cognition through the gut-brain axis.

## Conclusion

The molecular mechanisms underlying the effects of exercise on cognition are complex. Undoubtedly, brain plasticity is the direct target for exercise. In general, in both healthy individuals and patients with degenerative diseases, exercise can enhance brain plasticity through neurotrophic factors and synaptic plasticity. Neurotrophic factors nourish neurogenesis, as well as improving neuronal survival and regeneration in various regions, while synaptic plasticity is the basis for information transmission. For example, in mouse and human studies, the increase of hippocampal level of BDNF caused by exercise is proven to improve cognitive function; after exercise, both the increase of hippocampal volume and the expression of synaptic plasticity proteins will enhance LTP; and some studies have addressed the effect of exercise on adult neurogenesis, dendritic remodeling, and cognition. Mitochondria serve essential functions in neurons and mitochondrial maintenance directly affects neuronal development, function, and survival. Exercise maintains the stability of mitochondrial quantity and quality to some extent, improving the brain’s oxidative stress state and reducing the production of pro-inflammatory factors by microglia. Proteins involved in neuronal mitochondrial biogenesis, kinetics and autophagy, such as fusion proteins, cleavage proteins and proteins in the PINK1–Parkin pathway, may mediate the effects of exercise on neuronal mitochondria. Meanwhile, energy metabolism is critical to neuronal activity. Exercise maintains metabolism by reducing insulin resistance and increasing levels of CLUTs in the brain, providing energy basis for neuron activity.

The muscle-brain axis, the gut-brain axis and the liver-brain axis have been the focus and a hotspot in research of the mechanisms underlying exercise improvement of cognitive function in recent years. For example, the link between gut flora and brain cognition has now been continuously investigated. Fecal colony transplantation in aged mice with neurodegenerative diseases improves cognitive function, and the increase in AKK after exercise plays a role in cognitive improvement. However, other potentially beneficial or harmful gut flora and their products are still needed to be explored. Organs such as the liver and skeletal muscle have also attracted attention, as they have been demonstrated to produce factors that are involved in various physiological processes in the brain to maintain healthy cognitive function or inhibit pathological processes of various neurodegenerative diseases, ultimately delaying cognitive decline.

In the context of cognitive impairment in neurodegenerative diseases, there is often an abnormal accumulation of proteins. Excessive oxidative stress, energy metabolism disorder and neuroinflammation in the brain will aggravate this process. Therefore, exercise can not only improve neural plasticity, but also alleviate this abnormal pathological state. However, the effects may vary with individual genetic characteristics, disease type, exercise type, etc.

The pathways from exercise to cognitive function in the brain are vast and complex. Although significant progresses on molecular mechanisms have been made in recent years, more studies in greater depth are still needed. A full understanding of the mechanisms linking exercise to cognitive function can facilitate improvement of cognitive function in the elderly population, maintenance of the healthy cognitive status in the normal population, and development of drugs for cognitive function improvement.

## Data Availability

Not applicable.

## Abbreviations

MCI: Mild cognition impairment
MS: Multiple sclerosis
PD: Parkinson disease
NFT: Neurofibrillary tangle
BDNF: Brain-derived neurotrophic factor
CREB: CAMP-response element binding protein
PKC: Protein kinase C
TNF-α: Tumor necrosis factor-α
IL-1β: Interleukin-1β
QNP: Quiescent neural progenitor
INP: Intermediate neural progenitor
AHN: Adult hippocampus neurogenesis
LTP: Long-term potentiation
HD: Huntington disease
PGC-1α: Proliferator-activated receptor gamma co-activator 1-alpha
FNDC5: Fibronectin type III domain protein 5
TrkB: Tropomyosin-related kinase B
PI3K: Phosphatidylinositide 3-kinase
MAPK: Mitogen-activated protein kinase
PLCγ: Phospholipase Cγ
Akt: Protein kinase B
ERK: Extracellular signal-regulated protein kinase
BACE1: Beta-amyloid precursor protein cleaving enzyme 1
HDAC: Histone deacetylase
HAT: Histone acetylase
SIRT1: Sirtuin 1
NRF1/2: Nuclear respiratory factor 1/2
Tfam: Transcription factor A
Opa1/2: Optical atrophy 1/2
Mfn1/2: Mitofusin1/2
OMM: Outer mitochondrial membranes
Drp1: Dynamin-related protein 1
PINK1: PTEN-induced putative kinase 1
NAD: Nicotinamide adenine dinucleotide
PKA: Protein kinase A
TBI: Traumatic brain injury
PEDF: Pigment epithelium-derived factor
LIFR: Leukemia inhibitory factor receptor
CTSB: Cathepsin B
Gpld1: Glycosylphosphatidylinositol-specific phospholipase D1
GPI: Glycosylphosphatidylinositol
GLUT: Glucose transporter
GSK-3β: Glycogen synthase kinase-3β
mTOR: Mammalian target of rapamycin
TREM2: Myeloid 2
AKK: *Akkermansia muciniphila*
LPS: Lipopolysaccharide
TLR4: Toll-like receptor 4
MyD88: Myeloid differentiation primary response 88
NF-kB: Nuclear factor kappaB
